# Draft genome sequences of five *Escherichia coli* strains with human pathogenic attributes from animal reservoirs in the United States

**DOI:** 10.1128/mra.00736-26

**Published:** 2026-10-14

**Authors:** Aniket Sharma, Puja Boidya, Nicolas Blouin, Bledar Bisha

**Affiliations:** 1 Department of Animal Science, University of Wyoming 4416, Laramie, Wyoming, USA; 2 Department of Molecular Biology, University of Wyoming 4416, Laramie, Wyoming, USA; University of Maryland School of Medicine, Baltimore, Maryland, USA

**Keywords:** antimicrobial resistance, *Escherichia coli*, whole-genome sequencing

## Abstract

Both humans and animals harbor commensal *Escherichia coli* or potentially pathogenic and drug-resistant strains that can cause severe infections. Here, we report draft genomes of five multidrug-resistant *E. coli* isolates from raccoon, mouse, and cattle in the USA, revealing antimicrobial resistance, virulence-associated genes, and plasmid-associated determinants relevant to One Health.

## ANNOUNCEMENT

Antimicrobial resistance (AMR) is a silent pandemic and is projected to become a leading cause of global morbidity and mortality (1). Gram-negative bacteria, including *Escherichia coli,* are common members of the intestinal microbiota of humans and animals, but also cause nosocomial, foodborne, and waterborne infections (2–6). Many *E. coli* strains harbor AMR genes on mobile genetic elements (MGEs), facilitating transfer across hosts. Here, we report draft genome sequences of five multidrug-resistant (MDR) *E. coli* strains isolated in 2012 from raccoon, mouse, and cattle in the United States. Fecal samples were collected from animals, homogenized in universal pre-treatment broth, and plated onto MacConkey agar at 37°C for *E. coli* recovery. These genomes provide insight into AMR dissemination and highlight the role of animal-associated and agricultural populations in AMR ecology.

*E. coli* isolates were cultured on MacConkey agar (MilliporeSigma, USA) and incubated for 24 h at 37°C. Species identity was confirmed by matrix-assisted laser desorption/ionization-time-of-flight mass spectrometry (Bruker Ultraflex II, Biotyper RTC v3.1). Antimicrobial susceptibility testing was performed by broth microdilution using precoated plates containing 14 antibiotics (CMV3AGNF, Thermo Fisher Scientific, USA), and minimum inhibitory concentrations were determined after 18-24 h using SWIN v3.3 (Thermo Fisher Scientific, USA) and interpreted per CLSI guideline (7). MDR strains were selected for whole-genome sequencing.

Isolates were streaked on brain heart infusion agar (MilliporeSigma, USA) and incubated at 37°C for 24 h. A loopful of bacterial colony (10 µL) was used for DNA extraction with the DNeasy Blood & Tissue Kit (Qiagen, USA); DNA was extracted according to the manufacturer’s protocol and quantified using a Qubit 4.0 Fluorometer (Thermo Fisher Scientific, USA). Samples containing ≥100 ng of DNA (as per the manufacturer’s recommendation) were used for library preparation using the Illumina DNA Prep Kit (Illumina, USA). DNA was tagmented with bead-linked transposomes to generate fragments of ~350 bp, and dual i5/i7 indices were incorporated over five PCR cycles, followed by bead cleanup. We evaluated library quality and fragment size using a QIAxcel system (Qiagen, USA). Libraries were normalized, pooled, diluted to 750 pM in resuspension buffer with Tween-20, and sequenced on an Illumina NextSeq 2000 platform (P1 300-cycle cartridge) to generate 2 × 150 bp paired-end reads on a single flow cell. Sequencing yielded 1,161,399–2,496,779 reads corresponding to 71.5–149.4× genome coverage (Table 1).

**TABLE 1:** Sequencing summary, phenotypic and genomic characteristics of five *E. coli* isolates from animal sources in the US

Characteristics	Phenotypic, genomic, and resistome attributes				
Isolates	92	954	975	991	1178
Source	Raccoon	Cattle	Unknown	Cattle	Mouse
Sample type	Fecal	Fecal pat	Unknown	Fecal pat	Pellet
Draft genome size	4,750,064	4,631,235	5,039,138	4,924,731	4,696,289
GC content (%)	50.58	50.65	50.73	50.52	50.68
Total reads (paired end)	1,161,399	1,871,088	1,812,947	2,496,779	1,629,216
Genome coverage (×)	71.5	119.2	106.3	149.4	102.6
No. of contigs	116	110	134	119	107
Largest contig	409,155	278,605	572,970	290,142	409,166
N50/L50	127,781/12	122,863/14	180,850/10	182,061/11	121,687/13
Average contig length (bp)	53,978	53,851	62,989	62,338	55,908
Genes (including non-coding genes)	4,690	4,579	5,043	4,918	4,616
Coding sequences (total)	4,616	4,510	4,970	4,846	4,542
Predicted pathogenicity probability	0.935	0.83	0.89	0.88	0.94
Phylogroups	A	A	B1	C	A
Phenotypic resistance	Azithromycin, chloramphenicol, ciprofloxacin, nalidixic acid, streptomycin, tetracycline, trimethoprim/sulfamethoxazole	Amoxicillin/clavulanic acid, ampicillin, azithromycin, cefoxitin, ceftiofur, ceftriaxone, chloramphenicol, nalidixic acid, streptomycin, tetracycline	Azithromycin, ceftriaxone, ciprofloxacin, nalidixic acid, streptomycin	Amoxicillin/clavulanic acid, ampicillin, azithromycin, cefoxitin, ceftiofur, ceftriaxone, chloramphenicol, nalidixic acid, streptomycin, tetracycline	Amoxicillin/clavulanic acid, ampicillin, azithromycin, cefoxitin, ceftiofur, ceftriaxone, chloramphenicol, ciprofloxacin, nalidixic acid, streptomycin, tetracycline, trimethoprim/sulfamethoxazole
Intermediate resistance	None	None	Cefoxitin, ceftiofur, chloramphenicol, tetracycline	Ciprofloxacin	None
Predicted acquired ARGs	*mph(A)*, *floR*, *aadA1*, *aph(3″)-Ib*, *aph(6)-Id*, *tet(A)*, *sul2*, *sul3*, *dfrA17*, *mdfA*	*blaEC*, *blaHER-3*, *floR*, *tet(B)*, *blaEC-15*, *aph(6)-Id*, *blaTEM*	*tet(A)*, *sul2*, *blaEC-18*, *aph(6)-Id*, *aph(3″)-Ib*, *dfrA17*, *mph(A)*, *sul1*, *aadA5*, *blaTEM1*, *blaCMY-2*	*tet(A)*, *blaEC-13*	*aadA1*, *sul3*, *blaHER-3*, *floR, blaEC-18*, *blaEC-15*, *aph(3″)-Ib*, *dfrA17*, *mph(A)*, *sul1*, *blaTEM-1*, *dfrA8*
Predicted plasmid replicons	IncA/C2_1, Col(MG828)_1	IncA/C2_1	IncFIB(AP001918)_1	IncFIB(pHCM2) _1_ pHCM, IncI2_1_Delta	IncFIA_1, IncFIC(FII)_1
Predicted virulence genes	*espX1*, *espY1*, *espX4*, *espX5*, *ompA*, *iroE*, *entD*, *fepA*, *fes*, *entF*, *fepC*, *fepG*, *fepD*, *entS*, *fepB*, *entC*, *entE*, *entB*, *entA*, *csgG*, *csgF*, *csgD*, *csgB*, *aslA*, *yagV/ecpE*, *yagW/ecpD*, *yagX/ecpC*, *yagY/ecpB*, *yagZ/ecpA*, *ykgK/ecpR*, *fdeC*, *fimH*, *fimG*, *fimF*, *fimD*, *fimC*, *fimI*, *fimA*, *fimE*, *fimB*, *espL4*	*ybtS*, *ybtX*, *ybtQ*, *ybtP*, *ybtA*, *irp2*, *irp1*, *ybtU*, *ybtT*, *ybtE*, *fyuA*, *gspF*, *gspG*, *gspH*, *gspI*, *gspJ*, *gspK*, *espX4*, *espX5*, *espR1*, *ompA*, *espX1*, *csgB*, *csgD*, *csgF*, *csgG*, *astA*, *entD*, *fepA*, *fes*, *entF*, *fepC*, *fepG*, *fepD*, *entS*, *fepB*, *entC*, *entE*, *entB*, *entA*, *fimB*, *fimE*, *fimA*, *fimI*, *fimC*, *fimD*, *fimF*, *fimG*, *fimH*, *fdeC*, *ykgK/ecpR*, *yagZ/ecpA*	*gspM, gspL*, *gspK*, *gspJ*, *gspI*, *gspH*, *gspG*, *gspF*, *gspE*, *gspD*, *gspC*, *fimH*, *fimG*, *fimF*, *fimD*, *fimC*, *fimI*, *fimA*, *fimE*, *fimB*, *espX1*, *espX4*, *espX5*, *csgB*, *csgD*, *csgF*, *csgG*, *gtrB*, *entA*, *entB*, *entE*, *entC*, *fepB*, *entS*, *fepD*, *fepG*, *fepC*, *entF*, *fes*, *fepA*, *entD*, *yagV/ecpE*, *yagW/ecpD*, *yagX/ecpC*, *yagY/ecpB*, *yagZ/ecpA*, *ykgK/ecpR*, *fdeC*, *ompA*, *senB*	*entA*, *entB*, *entE*, *entC*, *fepB*, *entS*, *fepD*, *fepG*, *fepC*, *entF*, *fes*, *fepA*, *entD*, *espX5*, *yagV/ecpE*, *yagW/ecpD*, *yagX/ecpC*, *yagY/ecpB*, *yagZ/ecpA*, *ykgK/ecpR*, *fdeC*, *espX1*, *fimH*, *fimG*, *fimF*, *fimD*, *fimC*, *fimI*, *fimA*, *fimE*, *fimB*, *espL1*, *iucA*, *iucB*, *iucC*, *iucD, iutA, iroN, iroE*, *iroD*, *iroC*, *iroB*, *csgB*, *csgD*, *csgF*, *csgG*, *ompA*, *espR1*, *gspM*, *gspL*, *gspK*, *gspJ*, *gspI*, *gspH*, *gspG*, *gspF*, *gspE*	*espX1*, *espY1*, *ompA*, *entD*, *fepA*, *fes*, *entF*, *fepC*, *fepG*, *fepD*, *entS*, *fepB*, *entC*, *entE*, *entB*, *entA*, *espX5*, *csgG*, *csgF*, *csgD*, *csgB, aslA*, *espL4*, *espX4*, *fdeC*, *ykgK/ecpR*, *yagZ/ecpA*, *yagY/ecpB*, *yagX/ecpC*, *yagW/ecpD*, *yagV/ecpE*, *fimH*, *fimG*, *fimF*, *fimD*, *fimC*, *fimI*, *fimA*, *fimE*, *fimB*
BioSample ID	SAMN54754470	SAMN54754487	SAMN54754502	SAMN54754506	SAMN54754509
SRA ID	SRR37856037	SRR37856039	SRR37856024	SRR37856020	SRR37856017
Assembly accession numbers	GCA_056113005.1	GCA_056112365.1	GCA_056111905.1	GCA_056111685.1	GCA_056111615.1
Accession numbers	JBVYFG000000000	JBVYEQ000000000	JBVYED000000000	JBVYDZ000000000	JBVYDW000000000

Read quality was evaluated with FastQC v0.12.1 (8). Adapter sequences and low-quality bases were removed using Trimmomatic v0.39 (9), and filtered reads were assembled *de novo* with SPAdes v4.1.0 (10). Assemblies were annotated with the NCBI Prokaryotic Genome Annotation Pipeline v6.10 (11). Putative AMR and virulence genes were identified with ABRicate v0.2 (12) and VirulenceFinder v2 (13) using curated databases, respectively. Putative plasmid replicons were predicted with PlasmidFinder v2.0.1 (14) using default parameters. Additionally, all five isolates were predicted to be human pathogenic by PathogenFinder v2 (15).

These MDR, virulence genes, and plasmid replicons suggest opportunistic, pathogen-adapted lineages circulating across multiple animal reservoirs. These findings provide context on the role of wildlife and domestic animals as reservoirs and potential amplifiers of MDR *E. coli* and MGEs.

## Data Availability

Genome assemblies and raw sequencing reads have been deposited in the NCBI database under BioProject accession number PRJNA1405753. Individual BioSample, SRA, and accession numbers for each isolate are provided in Table 1.

## References

[1] Antimicrobial resistance: a silent pandemic. 2024. Nat Commun 15:6198. doi:10.1038/s41467-024-50457-z PMC11266683 39043632

[2] Boidya P, Ghorbani Tajani A, Blouin N, Bisha B. 2026. Draft genome sequence of multidrug-resistant *Raoultella ornithinolytica* isolated from a wastewater treatment plant in Laramie, Wyoming. Microbiol Resour Announc 15:e01051-25. doi:10.1128/mra.01051-25 PMC12797923 41395973

[3] Ghorbani Tajani A, Sharma A, Blouin N, Bisha B. 2024. Genome sequence, antibiotic resistance genes, and plasmids in a monophasic variant of *Salmonella typhimurium* isolated from retail pork. Microbiol Resour Announc 13:e00754-23. doi:10.1128/mra.00754-23 PMC11237804 38700351

[4] Ghorbani Tajani A, Sharma A, Ruehling K, Collins S, Bisha B. 2025. Molecular characterization of *Escherichia coli* isolate from the Greys-Hoback Watershed, Wyoming. Microbiol Resour Announc 14:e01187-24. doi:10.1128/mra.01187-24 PMC12424309 40741740

[5] Ghorbani Tajani Anahita, Hamidi A, Sharma A, Sylejmani D, Gorçaj E, Hashani G, Noci B, Bisha B. 2026. Draft genome sequence of multidrug-resistant *Escherichia coli* isolated from retail chicken liver in Kosovo. Microbiol Resour Announc 15:e01033-25. doi:10.1128/mra.01033-25 PMC12981117 41705850

[6] Mills EG, Martin MJ, Luo TL, Ong AC, Maybank R, Corey BW, Harless C, Preston LN, Rosado-Mendez JA, Preston SB, Kwak YI, Backlund MG, Bennett JW, Mc Gann PT, Lebreton F. 2022. A one-year genomic investigation of *Escherichia coli* epidemiology and nosocomial spread at a large US healthcare network. Genome Med 14:147. doi:10.1186/s13073-022-01150-7 PMC9801656 36585742

[7] Clinical and Laboratory Standards Institute (CLSI). 2025. EM100 connect-CLSI M100 ED35. EM100 connect. Available from: https://em100.edaptivedocs.net/GetDoc.aspx?doc=CLSI%20M100%20ED35:2025&xormat=SPDF&src=BB

[8] Andrews S. 2010. FastQC: a quality control tool for high throughput sequence data. http://www.bioinformatics.babraham.ac.uk/projects/fastqc.

[9] Bolger AM, Lohse M, Usadel B. 2014. Trimmomatic: a flexible trimmer for Illumina sequence data. Bioinformatics 30:2114–2120. doi:10.1093/bioinformatics/btu170 PMC4103590 24695404

[10] Prjibelski A, Antipov D, Meleshko D, Lapidus A, Korobeynikov A. 2020. Using SPAdes *de novo* assembler. CP in Bioinformatics 70:e102. doi:10.1002/cpbi.102 32559359

[11] Li W, O’Neill KR, Haft DH, DiCuccio M, Chetvernin V, Badretdin A, Coulouris G, Chitsaz F, Derbyshire MK, Durkin AS, Gonzales NR, Gwadz M, Lanczycki CJ, Song JS, Thanki N, Wang J, Yamashita RA, Yang M, Zheng C, Marchler-Bauer A, Thibaud-Nissen F. 2021. RefSeq: expanding the prokaryotic genome annotation pipeline reach with protein family model curation. Nucleic Acids Res 49:D1020–D1028. doi:10.1093/nar/gkaa1105 PMC7779008 33270901

[12] Seemann T. 2026. Tseemann/Abricate. Available from: https://github.com/tseemann/abricate

[13] Chen L, Yang J, Yu J, Yao Z, Sun L, Shen Y, Jin Q. 2005. VFDB: a reference database for bacterial virulence factors. Nucleic Acids Res 33:D325–D328. doi:10.1093/nar/gki008 PMC539962 15608208

[14] Carattoli A, Hasman H. 2020. PlasmidFinder and *in silico* pMLST: identification and typing of plasmid replicons in whole-genome sequencing (WGS). Methods Mol Biol 2075:285–294. doi:10.1007/978-1-4939-9877-7_20 31584170

[15] Ferrer Florensa A, Almagro Armenteros JJ, Kaas RS, Clausen PTLC, Nielsen H, Rost B, Aarestrup FM. 2026. Whole-genome prediction of bacterial pathogenic capacity on novel bacteria using protein language models with PathogenFinder2. Bioinformatics 42:btag129. doi:10.1093/bioinformatics/btag129 PMC13218381 41863303

